# Elimination of Bisphenol A and Triclosan Using the Enzymatic System of Autochthonous Colombian Forest Fungi

**DOI:** 10.5402/2013/968241

**Published:** 2012-09-18

**Authors:** Carolina Arboleda, H. Cabana, E. De Pril, J. Peter Jones, G. A. Jiménez, A. I. Mejía, S. N. Agathos, M. J. Penninckx

**Affiliations:** ^1^Biopolymers Group, Faculty of pharmaceutical Chemistry, University of Antioquia, Calle 67 No. 53-108, Antioquia, Colombia; ^2^Laboratory of Microbial Physiology and Ecology, Faculty of Sciences, Université Libre de Bruxelles, Institut de Santé Publique, rue Engeland 642, 1180 Brussels, Belgium; ^3^Unit of Bioengineering, Université Catholique de Louvain, Croix du Sud 2, 1348 Louvain-la-Neuve, Belgium; ^4^Department of Chemical Engineering, Université de Sherbrooke, 2500 boulevard de l'Université, Sherbrooke, QC, Canada J1K 2R1; ^5^Department of Civil Engineering, Université de Sherbrooke, 2500 boulevard de l'Université, Sherbrooke, QC, Canada J1K 2R1; ^6^Group of Taxonomy and Ecology of Colombian Fungi, Institute of Biology, University of Antioquia, Calle 67 No. 53-108, Antioquia, Colombia

## Abstract

Bisphenol A (BPA) and triclosan (TCS) are known or suspected potential endocrine disrupting chemicals (EDCs) which may pose a risk to human health and have an environmental impact. Enzyme preparations containing mainly laccases, obtained from *Ganoderma stipitatum* and *Lentinus swartzii*, two autochthonous Colombian forest white rot fungi (WRF), previously identified as high enzyme producers, were used to remove BPA and TCS from aqueous solutions. A Box-Behnken factorial design showed that pH, temperature, and duration of treatment were significant model terms for the elimination of BPA and TCS. Our results demonstrated that these EDCs were extensively removed from 5 mg L^−1^ solutions after a contact time of 6 hours. Ninety-four percent of TCS and 97.8% of BPA were removed with the enzyme solution from *G. stipitatum*; 83.2% of TCS and 88.2% of BPA were removed with the *L. swartzii* enzyme solution. After a 6-hour treatment with enzymes from *G. stipitatum* and *L. swartzii*, up to 90% of the estrogenic activity of BPA was lost, as shown by the yeast estrogen screen assay. 2,2-Azino-bis-(3-ethylthiazoline-6-sulfonate) (ABTS) was used as a mediator (laccase/mediator system) and significantly improved the laccase catalyzed elimination of BPA and TCS. The elimination of BPA in the absence of a mediator resulted in production of oligomers of molecular weights of 454, 680, and 906 amu as determined by mass spectra analysis. The elimination of TCS in the same conditions produced dimers, trimers, and tetramers of molecular weights of 574, 859, and 1146 amu. Ecotoxicological studies using *Daphnia pulex* to determine lethal concentration (LC_50_) showed an important reduction of the toxicity of BPA and TCS solutions after enzymatic treatments. Use of laccases emerges thus as a key alternative in the development of innovative wastewater treatment technologies. Moreover, the exploitation of local biodiversity appears as a potentially promising approach for identifying new efficient strains for biotechnological applications.

## 1. Introduction

Bisphenol A (BPA) and triclosan (TCS) are present in a great number of products of daily use. Residues of these products are found in many environmental matrices such as rivers and wastewater treatment plant effluents [1, 2]. BPA is a monomer or plasticizer in various polymeric materials [3], and residues of this compound in water appear to be due to incomplete polymerization [4, 5]. Triclosan (TCS) is used as a broad spectrum antibacterial agent and a preservative used in products of domestic use. It is found in cosmetics and personal care products such as shampoos, deodorants, and toothpastes and it also occurs in textile polymers and fibers to give them antibacterial properties [6]. The TCS structure strongly resembles to that of estrogens and dioxins [7]. Both of these compounds are frequently encountered in aquatic environments [8] and can incite serious risks for the environment and public health [9].

BPA has been recognized as an endocrine disrupting chemical (EDC). This chemical has the ability to imitate the female estrogen hormones which disrupt the body's chemical messenger system [10] due to its direct interaction with steroid receptors [11]. It has been argued that endocrine disruptors may be responsible for decline in sperm counts, abnormalities in the female reproductive tract, slow development in infants, increases in the incidence of testicular and breast cancer, and other medical disorders [12, 13]. The estrogenic effect of BPA is primarily linked to the presence of a core phenolic structure [14]. On the other hand, the chemical structure of TCS is related to many well-known xenobiotic compounds, such as halogenated diphenyl ethers and BPA. Little is known about the potential endocrine disrupting activities of TCS. Studies have shown that there are changes in fin length of the medaka fish *Oryzias latipes* exposed to TCS [15] suggesting that its metabolites can act like estrogen receptor antagonists [7]. TCS alone can alter the thyroid hormone receptor *α* transcript levels in the brain of premetamorphic tadpoles and induce a transient weight loss [16].

In recent years, the enzymatic degradation of lignin and environmental pollutants by the action of the phenoloxidase enzyme (laccase) has received increased attention [17–22]. White rot fungi (WRF) produce oxidative enzymes such as laccase, lignin, and manganese peroxidases, which are relatively nonspecific biocatalysts [21]. This versatile enzymatic system has been found to degrade a wide variety of environmental pollutants present in soil and water [23]. Few studies have used free ligninolytic enzymes to eliminate endocrine disruptors [20]. Crude and purified laccases (polyphenoloxidase, EC 1.10.3.2) were used to degrade BPA [19, 24–28] and TCS [19, 22, 24, 26]. 

The objectives of this work were to study the removal of BPA and TCS from aqueous solutions by using laccases excreted by the ligninolytic fungi *Ganoderma stipitatum* and* Lentinus swartzii* collected from the Colombian forest [29]. These WRF were selected as high laccase producers, after a preliminary screening of 30 WRF collected from a tropical forest in Colombia. Tropical forest constitute an important reservoir of microbial diversity including white rot fungi which have been identified as possible candidates for wastewaters treatment [21, 30, 31].

Specific aspects of this study included the utilization of a Box-Behnken-type factorial design to determine the effect of the experimental conditions (pH, temperature, and contact time) on their removal, the elimination of their estrogenic activity, and the potential improvement of the transformation of these chemicals in the presence of redox mediators. Finally, the products formed by the oxidative action of laccase on BPA and TCS were determined by mass spectra (MS) analysis and the acute toxicity of the liquid waste after treatment was tested by using the freshwater crustacean species *Daphnia pulex.*

## 2. Materials and Methods

### 2.1. Chemicals

Commercial grade reagents of the highest purity available were used in this work. All chemicals were from Sigma-Aldrich (St. Louis, MO, USA) and all solvents were of HPLC grade.

### 2.2. Organisms and Cultivation Conditions

The WRF strains *G. stipitatum *and* L. swartzii *were isolated from the Colombian tropical forest as previously reported [29]. The inocula were grown in Petri plates on malt extract agar 2% (w/v) at 25°C for about 10 days. Thereafter, the strains were maintained at 4°C until used. For enzyme preparation, liquid fermentation was performed on a orbital shaker at 150 rpm and 30°C in 250 mL Erlenmeyer containing 75 mL of standard medium: 10 g L^−1^ glucose, 0.2 g L^−1^ ammonium tartrate, 0.5 g L^−1^ Tween 80, 2.5 mM veratryl alcohol, 3 g L^−1^ tartaric acid, 0.2 g L^−1^ yeast extract, 0.5 mM CuSO_4_, and 0.8 g L^−1^ KH_2_PO_4_. The initial pH was adjusted to 5.0 with NaOH 2 M prior to autoclaving. The fermentation medium was inoculated with four mycelia agar plugs of 5 mm diameter. 

After 20 days of cultivation, the culture medium was filtered through a 0.22 *μ*m membrane filter (Millipore Corporation, Billerica, MA, USA). The filtrate was dialyzed against distilled water using a 14 kDa membrane (Spectrum Laboratories, Rancho Dominguez, CA, USA) and then used as the source of lignin modifying enzymes. Respective laccase production of *G. stipitatum *and* L. swartzii *was 11,000 and 9,000 U/L filtrate under these cultivation conditions [29].

### 2.3. Laccase Activity

Laccase activity was determined by monitoring the oxidation of 2,2′-azino-bis-(3-ethylbenzthiazoline-6-sulfonic acid) (ABTS) to its cation radical ABTS^●+^ at 420 nm (*ε*_max_ = 3.6 × 10 ^4^ M^−1^ cm^−1^) [32]. The assay mixture contained 0.5 mM ABTS. The pH was adjusted to 3.0 using 50 mM citrate/phosphate buffer and the temperature was set at 30°C. One unit (U) of activity was defined as the amount of enzyme forming 1 *μ*mol of ABTS^●+^ per min.

### 2.4. Enzymatic Treatment

The reaction mixture consisted of 5 mg L^−1^ of each of BPA (22 *μ*M) or TCS (18 *μ*M), 250 U L^−1^ of laccase activity, citric acid/disodium hydrogen phosphate buffer, and 1% v/v methanol. Prior to treatment, air was bubbled in the buffer solution overnight to saturate it with oxygen. The enzymatic treatments were performed in a 50 mL Erlenmeyer containing a 5 mL reaction media. The Erlenmeyer flasks were sealed with aluminum foil or paraffin film and placed in an orbital shaker at 150 rpm. 

To improve elimination of the two pollutants, some mediators were tested: ABTS, veratryl alcohol, 1-hydroxybenzotriazole (1-HBT), and guaiacol. Pollutant elimination incubations using mediators were carried out in 50 mL flasks with a reaction volume of 5 mL at pH 4 and 30°C for 1 hour, using a final mediator concentration of 10 *μ*M and a laccase concentration of 10 U L^−1^. The choice of a relatively low laccase concentration was made deliberately, because a too high laccase concentration could provoke a rapid and complete elimination of the pollutants and mask the potential effect of the mediators on the elimination of the selected micropollutants. Control flasks were prepared to quantify elimination in the absence of enzyme, mediator, or both. The enzymatic treatments were stopped by adding 3 drops of concentrated HCl (37% w/v). 

### 2.5. Extraction and Quantitative Analysis

BPA and TCS were extracted using ethyl acetate in a 1/1 volumetric ratio (ethyl acetate/treated solution). The solutions were acidified to pH 2 with HCl (37% w/v), shaken for 20 min, and then frozen overnight. The organic phase was separated and evaporated under a gentle stream of nitrogen. Each sample was dissolved in 100 *μ*L of methanol prior to quantitative chemical analysis.

The quantitative analysis of BPA and TCS was performed on an HPLC system consisting of a 600 controller, a 717 plus autosampler, and a 996 photodiode array detector (Waters, Milford, MA, USA). A Platinum EPS C_18_ 100A 5U 250 × 4.6 mm (Alltech, Deerfield, IL, USA) was used for the chromatographic separation. A 1 mL min^−1^ isocratic elution by means of 50% (A) acetonitrile with 10% of bidistilled water and 50% (B) phosphate buffer solution (10 mM KH_2_PO_4_, pH 3.2) with 10% acetonitrile was used to quantify the micropollutants at a wavelength of 277 nm.

### 2.6. Determination of the Estrogenic Activity by the YES-Assay

A recombinant yeast strain of *Saccharomyces cerevisiae *was used in a test (Yeast Estrogenic Screen, YES) designed to identify compounds interacting with the human estrogen receptor (hER) [33]. When the hER is bound to an estrogen-like compound, the receptor is coexpressed with the reporter gene *lac-Z*, which codes for the enzyme *β*-galactosidase. This enzyme is secreted into the medium and catalyzes the transformation of the chromogenic substance chlorophenol red-*β*-D-galactopyranoside (CPRG), which is subsequently measured colorimetrically in the medium. The absorbance resulting from the change of color from yellow to red is a direct measurement of the estrogenic activity of the compound tested [34]. The test was adapted to microtiter plates whose absorbance was read at 540 nm (for the color) and at 620 nm (for turbidity). The TCS was not subjected to this test because its microbicidal activity made the yeast test impracticable [19].

The positive wells were identified by a strong red coloring accompanied by the growth of the yeast. The estrogenic activity of the solution was correlated with the absorbance (*A*) of the solution by (1) as

(1) Acorrected=(A540  sample−A540  blank)−(A620 sample−A620 blank).

### 2.7. Identification of High Molecular Weight Reaction Products

To identify high molecular weight reaction products, a 5 mL mixture consisting of 5 mg L^−1^ of BPA or TCS, citric acid/disodium hydrogen phosphate buffer and 1% v/v methanol was used. The temperature was set at 30°C and the pH at 5.0. The concentration of laccase was 50 U L^−1^. The enzymatic reaction was allowed to take place for 24 h and it was stopped by acidifying the solution at a pH of 2 by adding 2 drops of HCL (37% w/v). The reaction mixture was centrifuged at 1,252 g for 90 min. The precipitate was dried under a gentle stream of nitrogen and suspended in 200 *μ*L of chloroform. This solution was subjected to mass spectra (MS) analysis. MS spectra were acquired using a Series 1200 LC-MS (Agilent Technologies, Santa Clara, CA, USA) equipped with an electrospray atmospheric pressure ionization (ESI) source in the negative ion monitoring mode, hereafter indicated as ESI(−). The ESI(−) conditions were set as follows: spray voltage, 4.5 kV; capillary voltage, 35 V; capillary temperature, 270°C; sheath gas, 17 U; collision gas pressure, 0.2 Pa. 

### 2.8. Acute Toxicity

Acute toxicity was assessed by bioassay using *Daphnia pulex*, according to a standard method [35]. Cultures were conducted under natural light conditions (1000–1500 lux) for a period of 16 h and 8 h under darkness at 22 ± 1°C and dissolved oxygen was kept over 80% of the saturation level. Samples were diluted into the culture media, at four different dilutions, 12.5, 25, 50, and 100%, representing the sample volume added/culture media volume used. The cultures were conducted under batch conditions throughout the test. The mean lethal concentration 50% (LC_50_) was determined after 48 h of culture as the dilution factor leading to the death of 50% of the microcrustaceans. 

### 2.9. Statistical Analysis

The impacts of the operational conditions pH, temperature, and contact time on the EDC-eliminations were determined by using a Box-Benhken-type factorial design [36]. The experimental conditions tested are presented in Table 1. The results presented in Table 2 were used for the determination of the regression coefficients of the second-order multiple regression model. The statistical analysis was performed using the Design Expert 6.0.11 software (Stat-Ease Inc., Minneapolis, MN, USA).

## 3. Results and Discussion

### 3.1. Effect of Temperature, pH, and Contact Time on the Enzymatic Elimination of BPA and TCS

To study the elimination of the two pollutants, three experimental parameters, pH, temperature, and processing time, were varied at three levels using the enzyme solutions of *G. stipitatum *or *L. swartzii* at a laccase activity final concentration of 250 U L^−1^. Coded values of pH, temperature, and time were followed using the factorial experimental design of Box-Behnken [36] shown in Table 1. 

This design was used in order to evaluate the impact of the three variables and of their potential interactions with each other upon the degradation of BPA and TCS, thus reducing the number of experiments required to test each parameter individually. Previous studies revealed that these parameters are operational variables with statistically significant influence on the performances of the biotreatment [19, 25, 26]. 

A multiregression model (see (2)) was developed to correlate the elimination of BPA and TCS by enzyme solutions produced by* G. stipitatum *or* L. swartzii *with the operational conditions by using a least squares method [37] as follows:

(2) Y=β0+∑i=1kβixi+∑i=1kβiixi2+∑i<j∑βijxixj+∑i<j∑βijxixj2,

where* Y* represents the predicted response (elimination of the EDC), *x* the *k*th variables (pH, temperature, and contact time), *β* are constant regression coefficients of the model, and *ε* is the random error component of the system.

Table 2 shows the considerable variation of elimination of the target compounds as a function of the operational parameters. The eliminations of BPA varied from 49.4 to 91.4% and from 8.3 to 81.6% for the laccases of *G. stipitatum *and* L. swartzii*, respectively. On the other hand, the TCS removal varied from 9.3 to 94.8% for the same laccase solutions depending of the operational conditions used. 

Tables 3(a) and 3(b) show the analysis of variance (ANOVA) for the transformation of the different EDCs by the laccases secreted by the two Colombian WRF strains tested. This table presents the statistically significant factors and interactions used for the development of the polynomial regression models. All of the models developed are statistically significant (*P* values < 0.05). These results are consistent with those obtained by Cabana et al. [19] using enzymatic solution from the WRF *Coriolopsis polyzona* and by Kim and Nicell [25, 26] with a commercially available *Trametes versicolor* laccase. 

The second order models for the respective elimination (*Y*) of BPA and TCS by the enzyme solutions from *L. swartzii *and* G. stipitatum* are presented through (3)–(5) considering only the significant terms determined by the ANOVA analysis (Table 3) as follows:

(3) YBPA,L.  swartzii  =−275.80+9.27X1+57.56X2+16.27X3−0.12X12−6.16X22−0.68X32+0.05X1X2+0.03X1X3−1.70X2X3,

(4) YBPA,G.  stipitatum=−244.33+109.93X1+0.50X  2+13.71X3−13.05X12−1.78X32+2.49X1X3,

(5) YTCS,G.  stipitatum  =−176.98+1.95X1+29.0X2    +36.69X3−6.72×10−3X12    −2.20X22−2.42X32+0.88X1X2−0.66X1X3−0.01X12X2  +5.98×10−3X12X3.

**Figure 1:**
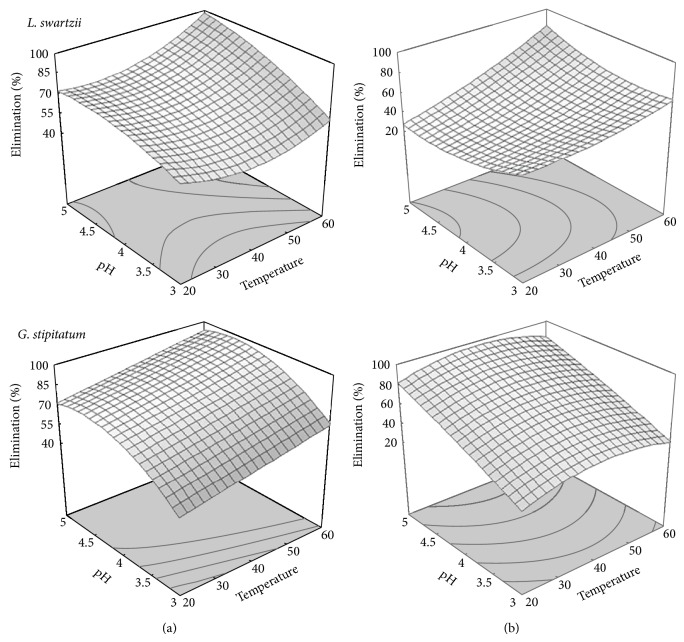
Response surface plots showing the effect of temperature and pH on the elimination after a 6 hour treatment of 5 mg L^−1^ of BPA (a) and 5 mg L^−1^ TCS (b) using 250 U L^−1^ laccase from *L. swartzii *and *G. stipitatum. *

The 3D surface responses obtained from these quadratic expressions are presented in Figure 1 in order to show the effects of the independent variables contact time, temperature, and pH and the interactive effects of each variable on the removal of the compounds of interest.

These quadratic models, based on a Box-Behnken experimental design, can be used to predict the optimal values of temperature and pH for the elimination of BPA and TCS among the range of values tested. Best temperatures were estimated to be between 40°C and 60°C for the two micropollutants and best pH was 5.0 except for a pH of 4.0 that was the best adapted for the elimination of BPA with the laccase from *L. swartzii*. The maximal extents of removal predicted by the models were obtained with laccase from *L. swartzii *for the 5 mg L^−1^ solution of BPA (97.5%, 60°C, pH = 5) and with laccase from *G. stipitatum* for TCS (93.9%, 40°C, pH = 5) when these enzymes were used at a level of 250 U L^−1^ for a contact time of 6 hours. These results are in agreement with a combination of stability produced by a higher pH and catalytic activity resulting from a higher temperature. An optimum pH of 3.0 for the initial rate of BPA removal by laccase from *Coriolus versicolor* was previously found [38], but the time course of the transformation of BPA under this pH condition was not reported. The optimum temperature and pH for the elimination of BPA using commercial laccase from *T. versicolor* were reported as 45°C and 5.0, respectively [25]. Finally, higher TCS removal by laccase of *C. polyzona* was observed at 50°C and pH of 5.0, whereas BPA removal culminated in a broad range between 40 and 50°C and pH of 5.0 [19].

### 3.2. Effects of Mediators

The use of low-molecular weight oxidizable substances in the biocatalytic cycle of laccase expands the activity of this enzyme. This mediated oxidation involves two oxidative steps. In the first one, the laccase oxidizes a primary substrate, the mediator, and this substance acts as an electron transferring compound. In the second step, the mediator transfers the electron from the substance of interest [39]. These mediators are known to increase the substrate range of laccase toward different substances [40]. Different mediators, namely, ABTS, veratryl alcohol, guaiacol, and 1-HBT, were tested for one hour at 30°C and pH 4 to degrade BPA and TCS, each present at initial concentration of 5 mg L^−1^ using laccases of *G. stipitatum *or* L. swartzii *at a final concentration of 10 U L^−1^. As a control, the buffer with the enzyme alone was used. The presence of the mediator alone did not allow elimination of BPA or TCS (results not shown).

**Figure 2:**
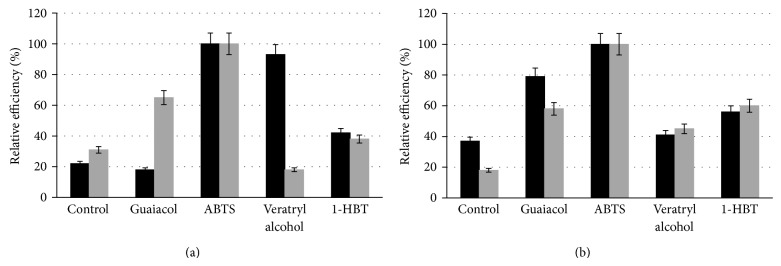
Effect of 10 *μ*M of different mediators on the removal of 5 mg L^−1^ of BPA (a) and 5 mg L^−1^ of TCS (b) after 1 h treatment at pH4.0 and 40°C with 10 U L^−1^ of laccase from (black square) G. *stipitatum *or (gray square)  *L. swartzii*. Control refers to the situation where mediator was absent.

Figure 2 presents the relative elimination of the BPA and TCS by the different laccase/mediator systems tested. A 100% relative efficiency refers to the removal of the EDC by the best combination of laccase and mediator. For both of these micropollutants, better extents of removal were obtained with ABTS for the two laccase solutions tested. This mediator helped to eliminate a higher percentage of micropollutants than that obtained with laccase alone (one-hour treatment at pH 4 and 30°C). The *G. stipitatum* and *L. swartzii* enzyme systems lacking a mediator had respective efficacy of 22 and 38% for removal of BPA, compared to the situation where ABTS was present (Figure 2(a)). Values of 38 and 18% were observed for the removal of TCS (Figure 2(b)). For the other mediators, guaiacol, veratryl alcohol, and 1-HBT, the improvement of the EDC transformation appeared to be also dependent from the nature of the enzymatic solution used (Figure 2). For example, guaiacol increased the removal of BPA when using laccase from *L. swartzii* whereas this conversion decreased when using laccase from *G. stipitatum* (Figure 2(a)). In contrast, guaiacol increased TCS removal when using laccase from *G. stipitatum* (Figure 2(b)). Veratryl alcohol (VA) strongly increased removal of BPA by *G. stipitatum*, but apparently had a slight decreasing effect on removal of this EDC by laccase of *L. swartzii* (Figure 2(a)). VA had a moderate increasing effect on TCS removal by both enzyme solutions (Figure 2(b)). Respective relative removal efficiency around 40 and 60% were observed for BPA and TCS by both enzyme solutions in the presence of 1-HBT (Figures 2(a) and 2(b)). The different behaviorof both enzymesshouldbe related totheirrespective responsivenesstowardsmediators, whichwas not studied here.

In conclusion, based on these results, ABTS was identified as the best mediator system for BPA and TCS elimination by laccases of all tested strains. This is consistent with the report by Cabana et al. [19] who found that the utilization of the laccase/ABTS system significantly improved the elimination of the EDCs nonylphenol, BPA, and TCS under the same operational conditions when using enzyme preparation excreted by *Coriolopsis polyzona* [19]. On the other hand, Tsutsumi et al. [28] determined that 1-HBT (0.2 mM) as a mediator could enhance BPA removal after one hour of treatment using 100 U L^−1^ of laccase from *Trametes versicolor*. In our own case, the same performance was obtained using a laccase/ABTS system but with only 10 U L^−1^ of laccase from any of the two new fungal strains described. Murugesan et al. [22] have observed that TCS was efficiently eliminated (up to 80%) by *Ganoderma lucidum* laccase in the presence of different mediators including, 1-HBT, ABTS, or syringaldehyde (SYD). Products of lower molecular weights than TCS including 2,4-dichlorophenol and dechlorinated forms were detected in the presence of 1-HBT or SYD. The radicals obtained from the oxidation of ABTS (2,2-azino-bis(3-ethylbenzothiazoline-6-sulfonate), in particular the radical cation ABTS^●+^, are quite stable [41] and acted as efficient mediators of laccase towards phenolics and some nonphenolic substrates, for example, Textile dyes [42] and polycyclic aromatic hydrocarbons [43]. There are, however, very few studies regarding the reaction of ABTS derived radicals with phenolic compounds [44, 45]. Yet, it seems that reaction schemes observed were not always compatible with a simple, phenol promoted, back reduction of ABTS derived radicals. Products obtained from triclosan in the presence of ABTS were not studied here and were apparently not reported in the literature [22, 26].

### 3.3. Identification of High Molecular Weight Reaction Products

In the absence of a redox mediator, BPA and TCS were transformed by laccase of *L. swartzii* and *G. stipitatum *into high molecular weight reaction products.

**Figure 3:**
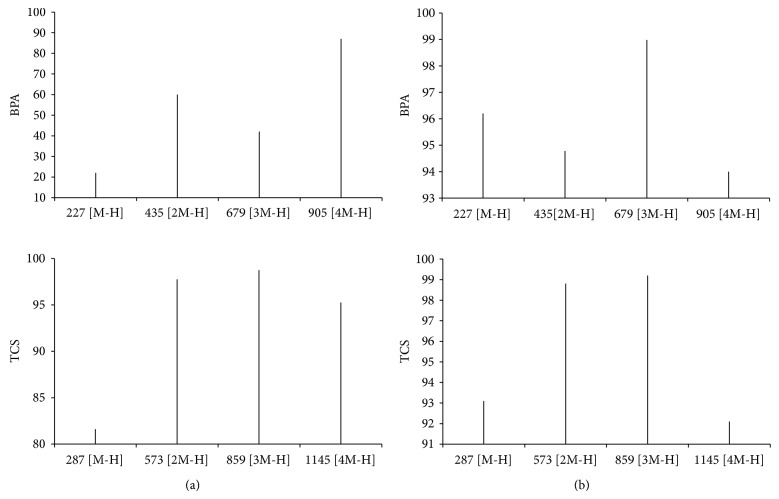
ESI(−)MS full scan spectrum of high molecular weight metabolites of BPA and TCS produced by action of the enzyme preparation containing 50 U L^−1^ of laccase from (a) *G. stipitatum* and (b) *L. swartzii* at a pH of 5.0 and a temperature of 30°C.

Figure 3 shows the ESI(−) MS spectra of chloroform-soluble reaction products of BPA produced by the enzymatic action of the two different enzyme sources. On these full ESI(−) MS spectra, *m/z* values of 227, 453, 679, and 905 represent, respectively, [M-H]^−^, [2M-H]^−^, [3M-H]^−^, and [4M-H]^−^ of BPA. The molecular weights of the BPA dimer, trimer and tetramer detected are, respectively, 454, 680, and 906 amu. 

Figure 3 also shows the ESI(−) MS spectra of chloroform-soluble metabolites of TCS produced by the enzymatic action of the enzymatic solutions. On these MS spectrum, *m/z* values of 287, 573, 859, 1145, and 1431 represent, respectively, [M-H]^−^, [2M-H]^−^, [3M-H]^−^, [4M-H]^−^, and [5M-H]^−^ of TCS. The molecular weights of these compounds are, respectively, 288, 574, 860, 1146, and 1432 amu. 

Huang and Weber [46] have shown that horseradish peroxidase transformed phenol-like chemicals via the formation of phenoxyl radicals. These radicals react with phenolic substances to form oligomers of the initial substrate. Cabana et al. [19] working with *Coriolopsis polyzona* laccase were the first to demonstrate that enzymatic treatment of nonylphenol (NP), bisphenol A (BPA), and triclosan (TCS) produced high molecular weight metabolites through a radical polymerization mechanism of NP, BPA, and TCS. This result was confirmed for TCS by Murugesan et al. [22] with *G. lucidum* laccase. Support for this polymerization mechanism with *L. swartzii* and *G. stipitatum* laccases comes from the ESI(−) MS spectra of BPA and TCS solutions treated with the enzyme solutions from both strains. The high molecular weight reaction products formed resulted from the oligomerization of oxidized products generated by laccase. ESI(−) MS spectra showed that this oligomerization could occur at the level of C–C or C–O bond formation. These types of bond could be between phenol moieties of BPA or TCS. This way of transformation has been proposed for BPA. The dimer produced from BPA by laccase of the WRF *Trametes villosa* was identified by NMR as 5,5′-bis-[1-(4-hydroxy-phenyl)-1-methyl-ethyl]-biphenyl-2,2′-diol [11]. Huang and Weber [46] also proposed possible reaction pathways in which the polymerization mechanism of BPA occurred through C–O bonds. Recently, Murugesan et al. [22] have shown that the utilization of the laccase/mediator system for TCS transformation results in the release of chlorine into the reaction media, while the utilization of free laccase did not result in the formation of dechlorinated products. These results suggested different reaction mechanisms for the transformation of TCS depending of the system used. In addition, Cabana et al. [47] identified TCS dechlorinated products generated by the utilization of laccase/chitosan conjugates for the treatment of TCS that is present in solution. These results indicated that the conditions used for the laccase-mediated oxidation of triclosan influenced the extent or the mechanisms of the reaction, which have an impact on the transformation products detected. It should be also noted that the differences between the products detected by these works can be explained by the different analytical methods used (single quadrupole (this work), triple quadrupole [19, 22], or quadrupole time-of-flight (QTOF) [47]). 

According to our results, the panel of reaction products associated with the elimination of BPA and TCS by the enzyme solution produced by the WRF tested appeared to be strain- and micropollutant-dependent. For example, laccase of *L. swartzii* was the most efficient for elimination of BPA, whereas laccase of *G. stipitatum* was the most effective for the removal of TCS. This is most probably due to different kinetic behavior (e.g., variation of the Michaelis-Menten kinetic parameters) as a function of strain and substrate that may strongly affect the extent of reaction and the metabolites produced [48]. 

### 3.4. Determination of Estrogenic Activity of the Treated BPA Solution by the YES Assay

**Figure 4:**
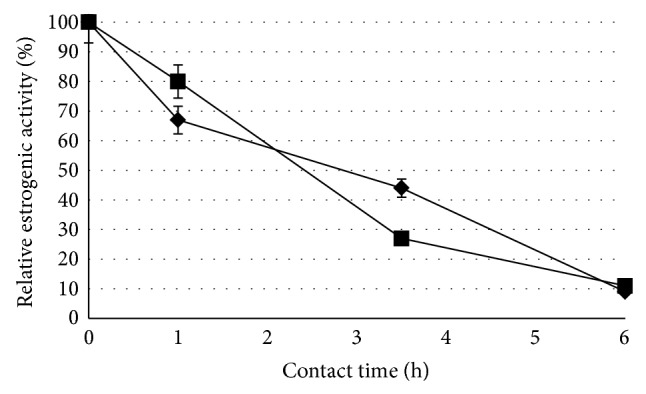
Elimination of the estrogenic activity of a 5 mg L^−1^ solution of BPA with the enzyme preparations from (black lozenge) * G. stipitatum *(pH 5 and 60°C) or (black square) *L. swartzii* (pH 4 and 40°C). The treatments were performed with a final concentration of laccase of 250 U L^−1^. Means of triplicates ± standard deviation.

Although the elimination of BPA and TCS was studied using the Box-Behnken factorial design, it was of great concern to address the elimination of the estrogenic activity associated with these compounds. To determine the estrogenic activity of the treated solutions, we used the well-established YES assay [33]. TCS was not subjected to this test because of its microbiocidal activity which makes this yeast test nonapplicable [19].  Figure 4 shows a clear estrogenic activity reduction observed after the enzymatic treatment of a 5 mg L^−1^BPA solution. Upon contacting this solution for 3.5 hours, with 250 U L^−1^ laccase from *L. swartzii*, there was a decrease of up to 70% in estrogenic activity and a 50% decrease with an equivalent treatment with laccase from *G. stipitatum*. Finally, after a 6 h treatment, estrogenicity loss of up to 90% of the BPA solution was observed for the laccases from both strains.

It had been previously observed that the estrogenic activity of a 0.88 mM solution of BPA was almost entirely removed by extending the laccase treatment to 12 h [28]. In our own experiments, the estrogenic activity of a 5 mg L^−1^ BPA solution was almost entirely removed after a laccase treatment of only 6 hours. The loss of estrogenic activity of BPA solution is apparently due to structural modification of the parent compound [19] as confirmed by the MS analysis (see Figure 3). The laccase mediated oxidation results in the formation of phenoxy radicals involved in the formation of BPA oligomers [12, 19, 46]. These products did not present the structural features necessary to bind to the hER [14]. Furthermore, the elimination of the estrogenic activity could be linked to the physical removal by precipitation of the polymers produced.

### 3.5. Elimination of the Acute Toxicity of the BPA and TCS by Enzymatic Treatments

The toxicities of the aqueous samples were measured using the microcrustacean* D. pulex* acute toxicity assay. Dilution factors used (0, 12.5, 25, 50, and 100%) to determine the acute toxicity represent the proportion of the BPA or TCS solution added to the culture media. The smaller the LC_50_ value, the more toxic is the tested solution.

Table 4 presents the LC_50_ determined for the solutions containing the initial 5 mg L^−1^ concentration of the micropollutants and for the treated solutions using 250 U L^−1^ of laccase activity from the different WRF strains at a pH of 5 and a temperature of 60°C. The initial BPA solution presented a LC_50_ value of 10% while the TCS solution had a LC_50_ < 12.5%. For both contaminants, the best toxicity reductions were obtained with the enzyme solution from the WRF strain *G. stipitatum*. In the analysis performed on samples from the BPA solution treated with that enzymatic solution, there was no observation of *D. pulex* death after the 48 hours incubation period at any of the dilutions tested (Table 4) which indicates that the LC_50_ is above the maximum concentration tested. On the other hand, the LC_50_ value of the TCS solution treated with this enzyme solution was 93%. It can be concluded that there was an important elimination of the toxicity associated with these contaminants after enzymatic conversion of BPA and TCS.

## 4. Conclusions 

In this paper, it was shown that among two newly described WRF strains producing laccases [29], the most efficient elimination of BPA was reached using the laccase of *L. swartzii *whereas for the removal of TCS the laccase of *G. stipitatum* was the most effective. Nevertheless, both strains were able to remove the estrogenic activities associated with BPA within a 6 h treatment. The elimination of the estrogenic activity of the BPA was associated with the formation of higher molecular weight reaction products. The MS spectra confirm the formation of oligomers (dimers through pentamers). It can also be concluded that the chemicals formed by the oxidative action of laccase presented a lower acute toxicity than the parent compounds. 

The biotreatment of wastewater using laccase-containing solutions secreted by WRF seems therefore to be an attractive solution for the elimination of micropollutants, including the known and suspected endocrine disruptors BPA and TCS. Laccases, which are regarded as environmentally friendly and relatively low-cost catalysts, may emerge as a key alternative in the development of new tertiary or polishing wastewater treatment technologies. The present contribution shows also that the exploitation of local biodiversity appears as potentially promising for identifying new efficient strains for biotechnological applications.

## Figures and Tables

**Table 1:** Level of variables chosen for the Box-Behken factorial design.

Variable	Symbol	Coded variable level		
		Low	Center	High
		−1	0	1
Temperature (°C)	*X*_1_	20	40	60
pH	*X*_2_	3	4	5
Contact time (hours)	*X*_3_	1	3.5	6

**Table 2:** Elimination of BPA and TCS from a 5 mg L^−1^ solution using an enzyme solution containing 250 U L^−1^ of laccase from the two strains of WRF tested for a contact time of 6 hours.

Coded variables		*G. stipitatum*		*L. swartzii *	
*X*_1_	*X*_2_	Elimination of BPA (%)	Elimination of TCS (%)	Elimination of BPA (%)	Elimination of TCS (%)
0	0	14.8	76.0	81.4	14.4
0	1	43.5	75.7	61.2	26.3
0	0	21.4	74.9	76.6	17.2
1	−1	11.7	51.0	25.1	23.4
0	1	71.1	94.8	81.6	59.8
0	−1	50.4	37.4	81.3	29.7
−1	1	29.8	73.3	22.0	29.3
0	−1	17.8	24.9	44.0	9.3
0	0	21.7	74.9	73.8	12.3
1	1	55.1	94.8	35.6	60.4
−1	0	68.9	60.9	36.5	25.1
0	0	22.3	75.5	77.3	11.7
1	0	84.2	90.1	47.9	49.7
1	0	55.3	53.6	13.3	25.3
0	0	27.1	78.9	77.8	27.6
−1	0	45.6	16.2	8.3	12.7
−1	−1	23.9	24.8	15.5	20.1

**Table:** (a)

BPA elimination							TCS elimination						
Source	Sum of square	Mean square	*F* value	*P* value	*R* ^2^	Coefficient of variation	Source	Sum of square	Mean square	*F* value	*P* value	*R* ^2^	Coefficient of variation
Statistical model	11953.8	1328.2	239.4	<0.0001	0.996	4.8	Statistical model	3799.5	422.2	14.3	0.001	0.905	23.1
*X*_1_	197.8	197.8	35.7	0.0006	—	—	*X*_1_	638.6	638.7	21.6	0.0023	—	—
*X*_2_	148.9	148.9	26.8	0.0013	—	—	*X*_2_	1089.2	1089.3	36.9	0.0005	—	—
*X*_3_	1816.5	1816.5	327.5	<0.0001	—	—	*X*_3_	1030.8	1030.8	34.9	0.0006	—	—
*X*_1_^2^	9173.5	9173.4	1653.9	<0.0001	—	—	*X*_1_^2^	193.5	193.5	6.6	0.0376	—	—
*X*_2_^2^	159.9	160.0	28.8	0.001	—	—	*X*_2_^2^	407.4	407.4	13.8	0.0075	—	—
*X*_3_^2^	75.1	75.1	13.5	0.0079	—	—	Interaction *X*_1_ *X*_2_	192.0	192.0	6.5	0.0381	—	—
Interaction *X*_2_ *X*_3_	72.1	72.1	13.0	0.0087	—	—	Residual error	206.7	29.5	—	—	—	—
Residual error	38.8	5.5	—	—	—	—							

Total	11992.7	—	—	—	—	—	Total	4006.3	—	—	—	—	—

**Table:** (b)

BPA elimination							TCS elimination						
Source	Sum of square	Mean square	*F* value	*P* value	*R* ^2^	Coefficient of variation	Source	Sum of square	Mean square	*F* value	*P* value	*R* ^2^	Coefficient of variation
Statistical model	10164.5	1694.1	113.3	<0.0001	0.9855	6.9	Statistical model	9200.6	920.0	221.8	<0.0001	0.992	5.3
*X*_1_	1621.7	1621.5	108.4	<0.0001	—	—	*X*_1_	920.2	920.2	221.8	<0.0001	—	—
*X*_2_	816.1	816.1	54.6	<0.0001	—	—	*X*_2_	3080.3	3080.3	742.5	<0.0001	—	—
*X*_3_	6255.2	6255.2	418.3	<0.0001	—	—	*X*_3_	207.4	207.4	50.0	0.0004	—	—
*X*_1_^2^	719.2	719.2	48.1	<0.0001	—	—	*X*_1_^2^	714.3	714.3	172.2	<0.0001	—	—
*X*_3_^2^	525.0	525.0	35.1	0.0001	—	—	*X*_3_^2^	960.0	960.0	231.4	<0.0001	—	—
Interaction *X*_1_ *X*_3_	155.0	155.0	10.4	0.0092	—	—	Interaction *X*_1_ *X*_3_	336.7	336.7	81.2	0.0001	—	—
Residual error	149.6	15.0	—	—	—	—	Interaction *X*_1_^2^ *X*_2_	43.7	43.7	10.5	0.0176	—	—
							Interaction *X*_1_^2^ *X*_3_	71.4	71.4	17.2	0.006	—	—
							Residual error	24.9	4.1	—	—	—	—

Total	10314.1	—	—	—	—	—	Total	9225.5	—	—	—	—	—

**Table 4:** Acute toxicity of the untreated and treated solutions containing initially 5 mg L^−1^ of BPA and TCS by using *D. pulex. *The enzymatic treatments occur for 6 hours at a pH of 5 and a temperature of 60°C using 250 U L^−1^ of laccase activity.

	Dilution factor used (%)	Number of dead organisms	Mortality (%)	LC_50_% (equivalent dilution factor)	Dilution factor used (%)	Number of dead organisms	Mortality (%)	LC_50_% (equivalent dilution factor)
Control (buffer)	0	0	0	>100	—	—	—	—
	12.5	0	0		—	—	—	—
	25	0	0		—	—	—	—
	50	0	0		—	—	—	—
	100	0	0		—	—	—	—
	BPA				TCS			
Solution before the enzymatic treatment	0	0	0	10	0	1	5	<12.5
	12.5	15	75		12.5	20	100	
	25	20	100		25	20	100	
	50	20	100		50	20	100	
	100	20	100		100	20	100	

Treated solution by the enzymatic solution from								
*L. swartzii *	0	0	0	>100	0	0	0	24
	12.5	1	5		12.5	6	30	
	25	0	0		25	7	35	
	50	2	10		50	20	100	
	100	1	5		100	20	100	
*G. stipitatum *	0	0	0	>100	0	0	0	93
	12.5	0	0		12.5	0	0	
	25	0	0		25	0	0	
	50	0	0		50	0	0	
	100	0	0		100	12	60	
